# Strategies for selecting perforator vessels for transverse and oblique DIEP flap in male pediatric patients: Anatomical study and clinical applications

**DOI:** 10.3389/fped.2022.978481

**Published:** 2022-09-23

**Authors:** Jiqiang He, Huairui Cui, Liming Qing, Panfeng Wu, Gunel Guliyeva, Fang Yu, Maolin Tang, Juyu Tang

**Affiliations:** ^1^Department of Hand and Microsurgery, Xiangya Hospital of Central South University, Changsha, Hunan, China; ^2^Department of Anatomy, School of Basic Medical Sciences, Wenzhou Medical University, Wenzhou, China; ^3^Division of Plastic Surgery, Mayo Clinic, Jacksonville, FL, United States

**Keywords:** lower extremity, children, soft-tissue defects, deep inferior epigastric perforator flap, reconstruction

## Abstract

**Background:**

Transverse and oblique deep inferior epigastric artery perforator (DIEP) flaps are widely used in breast, lower extremity, urogenital, head and neck reconstruction. In this report, we present our experience with selecting perforator vessels for transverse and oblique DIEP flaps based on an anatomical study and clinical cases.

**Materials and methods:**

A detailed anatomical study of the DIEP flap was carried out using a standardized injection of lead oxide in 10 fresh cadavers. Additionally, 35 male pediatric patients (age 5–12 years) underwent lower extremity reconstruction with a DIEP flap. A transverse DIEP flap was used when the defect template did not exceed zone IV, while an oblique DIEP flap was used when the defect template exceeded zone IV.

**Results:**

Perforators located below the umbilicus in zones I and II were rich in transverse anastomoses across the midline of the abdominal wall, which is the basis for the transverse DIEP flap. Perforators lateral to the umbilicus in zone I had true anastomoses with the musculophrenic artery, the morphological basis for the oblique DIEP flap. The DIEP flap design was transverse in 20 patients and oblique in 15. Flap sizes ranged from 8 × 4.5 cm^2^ to 24 × 9 cm^2^. One oblique DIEP flap was necrosed totally, and it was repaired by a latissimus dorsi musculocutaneous flap.

**Conclusion:**

The transverse DIEP flap design based on the perforator located below the umbilicus in zone I is recommended for small skin and soft tissue defects. We recommend the use of the oblique DIEP flap design based on the perforator lateral to the umbilicus in zone I as an extended flap to reconstruct large tissue defects.

## Introduction

The deep inferior epigastric perforator (DIEP) flap, first described by Koshima and Soeda in 1989 as an inferior epigastric artery skin flap, has become one of the most popular perforator flaps and the gold standard for free autologous tissue breast reconstruction (1). The DIEP flap provides autologous tissue for breast reconstruction without sacrificing the underlying rectus muscle, reducing the risk of bulge or hernia formation (2, 3). Due to the advantages mentioned above, the DIEP flap is also used for reconstruction of the head and neck, lower extremity, and urogenital areas (4–7). However, the conventional transverse design of the DIEP flap has poor perfusion in zone IV, which limits the flap size (8, 9). Though the transverse DIEP flap size can be extended using a pre-expansion procedure or anastomosis with another vessel, these procedures are time-consuming and surgically complex (10, 11). In those cases, the oblique DIEP flap can be used (12).

The use of the transverse and oblique DIEP flaps for reconstruction §of soft-tissue defects in the extremities of pediatric patients has been reported by other authors and us (13–15). Nonetheless, the optimal method for choosing suitable perforators for the transverse and oblique DIEP flap designs has not been widely reported. Therefore, the purpose of the present study was to describe our method of choosing different perforators for the transverse and oblique DIEP flap designs based on the results of an anatomical study and clinical cases.

## Materials and methods

### Anatomical study

This project was approved by the Institutional Health Sciences Human Ethics Committee. Whole-body injections were administered to the fresh cadavers following a protocol similar to that described previously (16). Ten cadavers (8 males and 2 females) were included in this study, mean age, 30 years [range, 25–40 years]; mean height, 167 cm [range, 161–177 cm]). All the cadavers were obtained through the Xiangya School of Medicine Donor Program. Abdomens were dissected to study the vascularization and composition of the DIEP flap. For this purpose, plain radiography, computed tomography, three-dimensional (3D) reconstruction [Mimics software (17)], and hierarchical anatomy were used. In addition, the locations, source, number, and extended anastomosis of the skin arteries were studied.

### Patients

The surgical procedures were conducted in our department. This study followed Ethical Committee guidelines of our institution, and the protocol was developed in accordance with the ethical standards of the Helsinki Declaration of 1975 and all subsequent revisions. Written informed consent was obtained from the [individual(s) AND/OR minor(s)' legal guardian/next of kin] for the publication of any potentially identifiable images or data included in this article. Inclusion criteria: (i) patients younger than 14 years of age; (ii) no injury history in donor sites; (iii) the guardians of the patients agreed to receive one-stage reconstructive surgery with a free DIEP flap. Exclusion criteria: (i) lost-to-follow up patients; (ii) patients with serious underlying/concomitant diseases; (iii) patients with previous flap surgery.

Between January 2004 and December 2020, 35 boys aged 5 to 12 years (mean: 7 years) underwent lower extremity reconstruction with free DIEP flap. All the wounds were caused by trauma. The defect size ranged from 7 × 4 cm^2^ to 24 × 8 cm^2^, and 12 patients had concomitant fractures. The demographic data, wound etiology, wound location, outcomes, complications, and follow-up results are shown in Table 1.

**Table 1:** Demographic data of patients who underwent DIEP.

**Patient characteristics**	**No**.
No.	35
Age (years)	7 (5 to 12 years)
Demographics	
Male	35
Cause	
Traumatic	35
Location	
Lower leg	4
Ankle and foot	31
Skin defects (cm^2^)	7 × 4 to 24 × 8
Flap design	
Transverse	20
Oblique	15
Recipient vessels	
Anterior tibial artery	2
Posterior tibial artery	23
Dorsalis pedis artery	10
Complications	
Total flap necrosis	1
Flap bulky	14
Hypertrophic scars	6
Follow-up period (months)	19.8 (6 to 72)

DIEP, deep inferior epigastric artery perforator flap.

### Surgical technique

Thorough initial debridement was conducted before the patients were sent to our institution for subsequent reconstruction. All patients received negative pressure wound therapy. The perforators were detected and marked preoperatively with a handheld Doppler (18, 19). The transverse or oblique DIEP flap design was selected based on the size of the defect template. If the defect template did not exceed zone IV, a transverse design based on the perforator located below the umbilicus in zone I was selected. If the defect template exceeded zone IV, an oblique DIEP flap was designed using the perforators lateral to the umbilicus in zone I.

DIEP flap harvest was performed as previously described (15, 20). The flap was raised from lateral to medial until the target perforators were identified. Retrograde dissection of the perforator was carefully performed, and the muscular vessel branches were coagulated or clipped to avoid bleeding. The motor nerve of the rectus abdominis was preserved as much as possible. The deep inferior epigastric vessels (DIEV) were dissected through the anterior rectus fascia and rectus abdominis until the appropriate pedicle length and diameter were achieved. The flap was then transferred to the recipient area and the DIEV were anastomosed with the vessels at the recipient site in an end-to-side or end-to-end manner. The donor site was primarily closed.

Postoperatively, the extremities were kept warm and elevated. Postoperative monitoring was constituted of hourly flap checks to evaluate the color, capillary refill, turgor, and surface temperature. Patients also received multimodal pain management and appropriate antibiotics in accordance with wound microbiological cultures.

## Results

### Anatomical study

**Figure 1:**
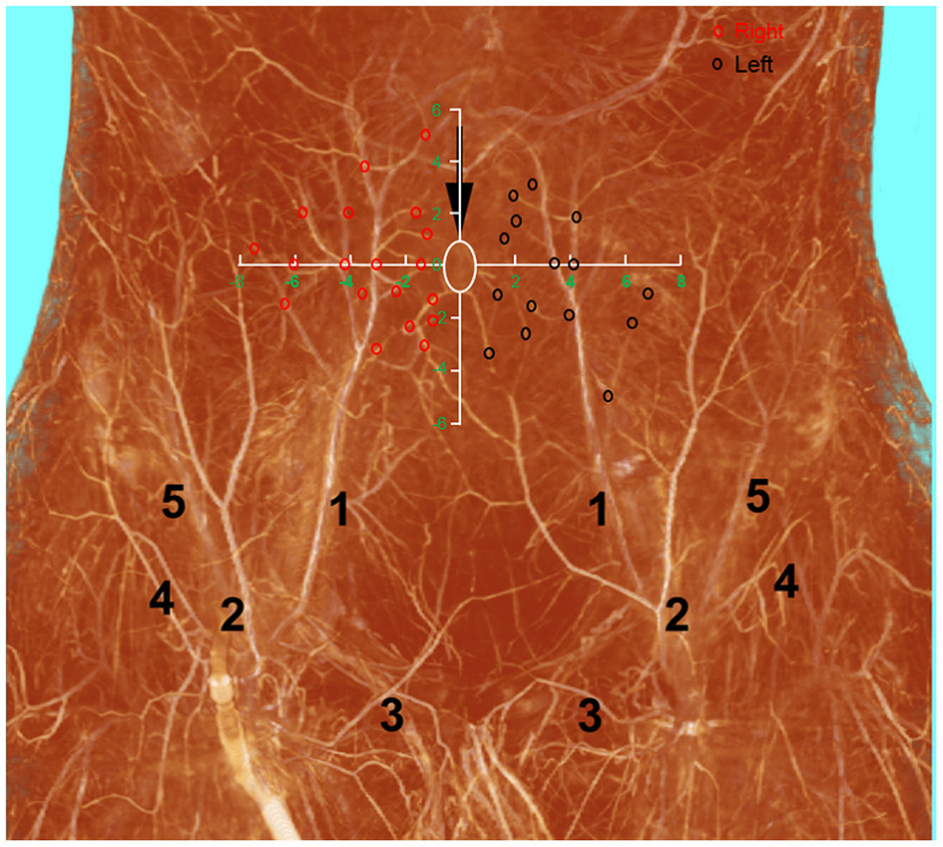
A diagram describing the distribution of DIEP perforators drawed on the three-dimensional visualization of the abdominal wall of a male cadaver after angiographic injection. DIEP, deep inferior epigastric perforator; white ellipse, umbilicus, red and black circles–DIEP perforator locations; 1, deep inferior epigastric artery; 2, superficial inferior epigastric artery; 3, superficial external pudendal artery; 4, superficial iliac circumflex artery; 5, deep iliac circumflex artery.

**Figure 2:**
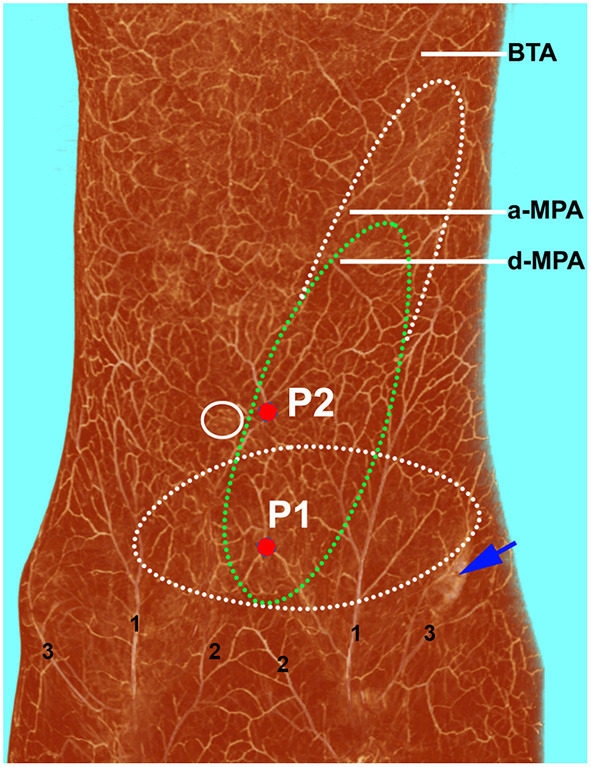
Three-dimensional visualization of the abdominal wall of a female cadaver after angiographic injection. The perforasome of the DIEP flap are linked to many other perforasome *via* “choke vessels” in the subdermal plexus. The transverse (white dotted ellipse) and oblique (green dotted ellipse) DIEP flap designs can be safely harvested using perforator 1 (P1). If perforator 2 (P2) is chosen, an oblique extended DIEP flap can also be safely harvested, as the distal part of the flap is also composed of dynamic territories. DIEP, deep inferior epigastric perforator; BTA, brachiothoracic artery; a-MPA, ascending branch of the musculophrenic artery; d-MPA, descending branch of the musculophrenic artery; 1, superficial inferior epigastric artery; 2, superficial external pudendal artery; 3, superficial iliac circumflex artery.

The deep inferior epigastric artery originated from the external iliac artery before running upward along the inner margin of the deep inguinal ring in the extraperitoneal tissue. The deep inferior epigastric artery then penetrated the thin layer of the transversus abdominis fascia into the deep surface of the rectus abdominis and then usually divided into medial and lateral branches. These branches provided the medial and lateral row perforators that supplied the abdominal fat and skin. The medial row perforators were the dominant arteries of the DIEP, which were located in the medial third of the rectus abdominis. The lateral row perforators were located in the lateral third of the rectus abdominus. These perforators were mainly distributed within a distance of 8 cm horizontally and 8 cm vertically from the umbilicus, especially within a radius of 4.0 cm around the umbilicus. The 3D reconstruction showed that the perforasome of the DIEP flap linked to many other perforasome *via* “choke vessels” in the subdermal plexus. Perforators located below the umbilicus in the zone I and II were rich in transverse anastomoses across the midline of the abdominal wall (Figure 1). These true transverse anastomoses comprise the critical morphological basis for the transverse DIEP flap. Moreover, perforators lateral to the umbilicus (P2) in the zone I had true anastomoses with the musculophrenic artery (Figure 2). The distal part of the oblique DIEP flap was composed of dynamic territories. Thus, the extended DIEP flap could be safely harvested.

### Clinical cases

In this series, a transverse DIEP flap design was used in 20 patients, and an oblique design was chosen for the rest. The donor site was closed primarily in all patients. Flap sizes ranged from 8 × 4.5 cm^2^ to 24 × 9 cm^2^. The DIEV was anastomosed to the recipient's vessels either end-to-side or end-to-end. The recipient's vessels were the anterior tibial artery in 2 cases, the posterior tibial artery in 23 cases, and dorsalis pedis artery in 10 cases. The follow-up period ranged from 6 to 72 months, with a mean follow-up of 19.8 months. The wounds healed well. DIEP flap provided reliable soft tissue coverage and good contour. All 12 patients with fractures achieved union. All patients were able to ambulate independently at the final follow-up. No patient developed late wound complications, signs of hernia, or breakdown within the follow-up period.

Nonetheless, one oblique flap became totally necrosed due to the arterial thrombosis, and it was repaired using a latissimus dorsi musculocutaneous flap. Venous thrombosis of one transverse flap resulted in the partial loss of the flap after salvage procedure. Fourteen patients experienced bulky flaps that required a defatting procedure. As expected, the donor site scar was more obvious in patients treated with the oblique flap than those treated with the transverse flap. Six kids developed hypertrophic scars (1 transverse DIEP, 5 oblique DIEP). To improve the scar appearance, local flaps and surgical techniques such as Z-plasty were used.

### Case reports

#### Case 1

**Figure 3:**
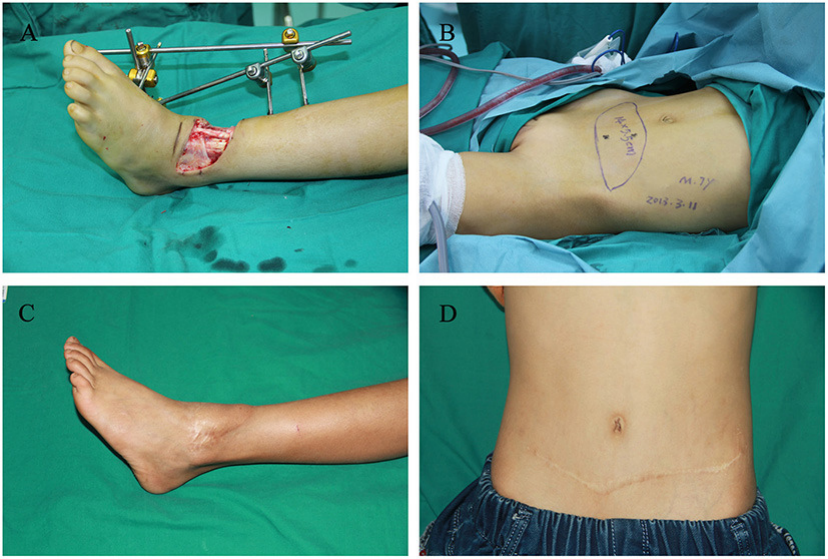
Images of the 7-year-old boy in case 1. **(A)** Left ankle soft tissue defect caused by a car accident. **(B)** Transverse deep inferior epigastric perforator flap design. **(C)** Recipient site at 30 months postoperatively. **(D)** Donor site at 30 months postoperatively.

Case 1 was a 7-year-old boy with 13 × 4 cm^2^ final skin and soft tissue defect who presented the day after a car accident (Figure 3A). A 14 × 5.5 cm^2^ transverse DIEP flap was harvested and no flap necrosis was observed (Figures 3B,C). Thirty months postoperatively, the patient had full ankle mobility and a slightly noticeable linear scar on the abdominal wall (Figure 3D) (Supplementary Video 1).

#### Case 2

**Figure 4:**
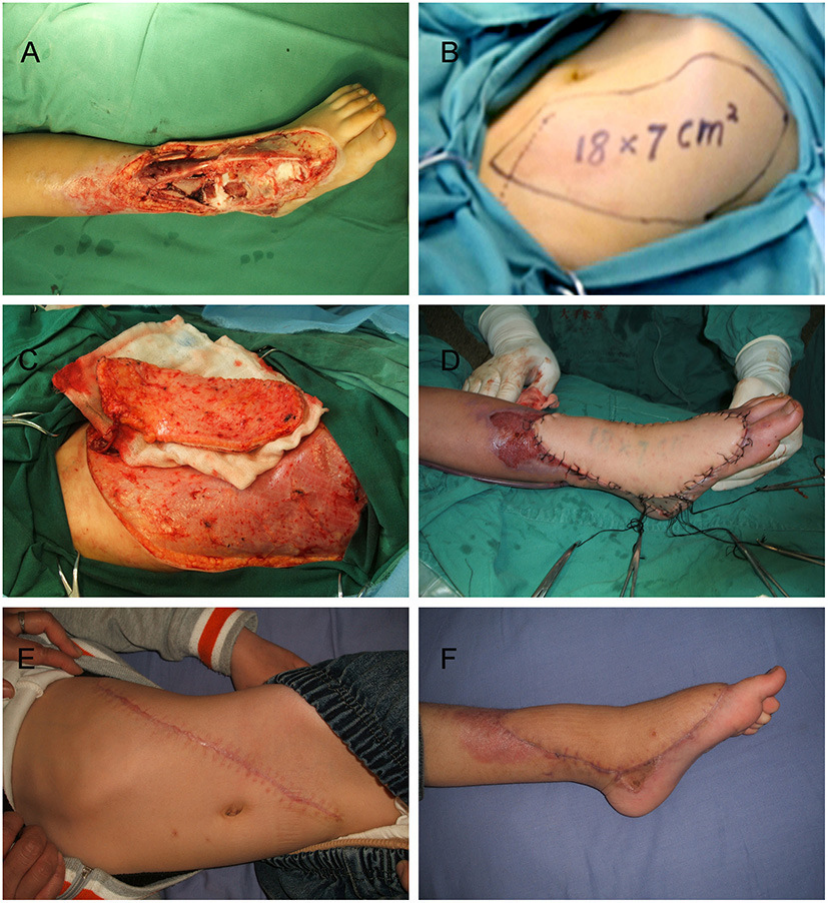
Images of the 8-year-old boy in case 2. **(A)** Large soft tissue defects on the left foot and ankle. **(B)** Oblique deep inferior epigastric perforator flap design. **(C)** Harvest of the flap. **(D)** Inset of the flap. **(E)** Donor site at 5 years postoperatively. **(F)** Recipient site at 5 years postoperatively.

Case 2 is an 8-year-old boy who presented with a large wound in his left leg after radical debridement suffered in a motor vehicle accident (Figure 4A). An 18 × 7 cm^2^ oblique DIEP flap was harvested and transferred (Figures 4B–D) At the 5-year follow-up, there was a relatively obvious oblique scar on the abdominal wall (Figure 4E), but the flap had good contour in the reconstructed areas (Figure 4F). The gait and shoe-wear were both normal. The patient was able to ambulate without assistance and had a good ankle functional recovery (Supplementary Video 2).

## Discussion

Free flaps are considered to be the gold standard for lower extremity soft tissue reconstruction. Among them the anterolateral thigh flap (ALT) has the advantage of providing thin, pliable skin in addition to having low donor site morbidity (21). However, in some cases, ALT cannot be harvested, or the size of the ALT is limited (in young males due to the lower elasticity of the thigh envelope) (15, 22). The thoracodorsal artery perforator flap and latissimus dorsi muscle flap are versatile in lower extremity reconstruction, as well (23, 24). However, donor site morbidity and intraoperative position change need to be taken into consideration. On the other hand, the gracilis flap is easy to harvest and has low donor site morbidity. More importantly, the denervated gracilis muscle flap will atrophy later, thus achieving improved flap contour and aesthetic outcomes, which makes it ideal for use in children (25, 26). Nonetheless, the gracilis flap requires a split-thickness skin graft to cover the muscle flap, which causes damage to a second donor site. Moreover, the size of this flap does not allow the reconstruction of the large defects.

In the cases mentioned above, the DIEP flap can be a reliable alternative. The abdomen is an ideal donor area because it has well-defined vascular anatomy, an abundance of perforating arteries, and it is generally concealed (27–29). Furthermore, the large surface area of the abdomen makes it a suitable donor site for the reconstruction of large defectsand allows two teams to work on the patient concurrently (one at the donor site and one at the defect site). Both transverse and oblique DIEP flaps are reportedly reliable for soft tissue reconstruction (12, 30). However, many studies have shown that the transverse DIEP flap design has poor perfusion in zone IV (31, 32). Therefore, partial necrosis may occur if the flap harvest exceeds zone IV. To prevent this complication, pre-expansion procedure, double venous system anastomosis, and extra harvest of the superficial inferior epigastric artery (SIEA) to perfuse the flap can be used (33–36). However, these methods are time-consuming and surgically complex, which might limit their use.

**Figure 5:**
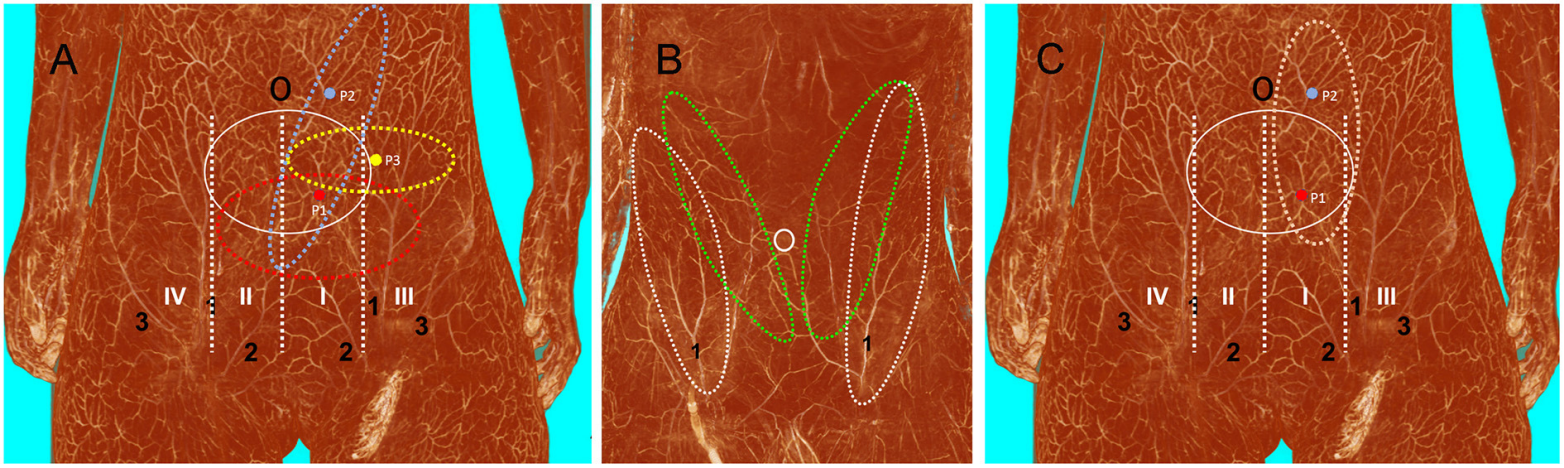
Three-dimensional visualization of the abdominal wall. **(A)** Blood supply (white solid ellipse) from the deep inferior epigastric artery in a female cadaver after angiographic injection. If the perforator is located in zone I (P1), tissue from zones I, II, and III can be safely harvested using the transverse design (red dotted ellipse). If the perforator is located in zone III (P3), only tissue from zones I and III can be safely harvested using the transverse design (yellow dotted ellipse). Both P1 and perforator 2 (P2) can be used to harvest the oblique DIEP flap, but using P2 enables the harvest of a large amount of tissue with P2 at the center of the flap (blue dotted ellipse). **(B)** Anatomical basis of the oblique DIEP flap (green dotted ellipse) and SIEA flap (white dotted ellipse) in a male cadaver after angiographic injection. **(C)** Anatomical basis of the vertical DIEP flap. DIEP, deep inferior epigastric perforator; SIEA, superficial inferior epigastric artery; 1, superficial inferior epigastric artery; 2, superficial external pudendal artery; 3, superficial iliac circumflex artery.

In addition to the oblique and transverse designs for the DIEP flap, a third vertical design based on the perforator angiosome (Figure 5C) was proposed by Tan et al. in 2009 (37). They reported that the vertical incision shortens the operation time and avoids the problem of poor perfusion in the distal part of the flap. Furthermore, the vertical flap can be individually harvested in accordance with the wound size, and the donor site can be closed directly. Unfortunately, we do not have much experience with the vertical DIEP flap design in our department.

The abdominal wall has abundant perforators, and it remains unclear how to choose the most suitable perforators for the transverse and oblique DIEP flap designs. The handheld doppler has been routinely used to locate the perforators. But significant inconsistencies between pre-operative and intra-operative findings have been reported (38). Multi-slice CTA is the current gold standard for perforator mapping. However, the disadvantages of CTA should be considered, such as radiation exposure, need for IV access and risks of contrast administration (39). According to the present anatomical study, the locations of the perforators influence the outcome of flap surgery. If the perforator is located in zone I (P1), it is safe to harvest tissue in zones I, II, and III. However, if the perforator is located in zone III (P3), it is only safe to harvest tissue in zone II and III (Figure 5A). Thus, in that case, it is not possible to harvest a flap that crosses the midline of the abdominal wall (32). Either P1 or P2 can be used for the oblique DIEP flap design, but the largest oblique DIEP flap can be harvested if the perforator is located in the zone I parallel to the umbilicus (P2). Additionally, as reported by Miyamoto et al., a perforator from the SIEA can be used in the oblique flap design (40) (Figure 5B). According to our experience and anatomical findings, we recommend choosing the perforators below the umbilicus in zone I when harvesting the transverse DIEP flap, while perforators lateral to the umbilicus in zone I were chosen when harvesting the oblique DIEP flap. The results of the present anatomical study show that the oblique design of the DIEP flap can be safely harvested without poor perfusion of the distal part, which expands the sizes of DIEP flaps to repair large skin and soft tissue defects (41). In all of our cases, DIEP flaps were successfully harvested and transferred without significant poor perfusion in the distal part of the flap. However, one should keep in mind that both the oblique and transverse DIEP flap designs have certain disadvantages. First, we do not recommend using it for girls, which precludes the use of DIEP flap for future breast reconstruction. Second, in our study, the rate of bulky DIEP flaps that required a secondary defatting procedure is high (14/35, 40%) (42). Third, both the oblique and transverse designs leave scars in the abdominal area, which is more prominent with the oblique design.

## Limitations

This study is a descriptive retrospective case series with limited number (35) of patients and no comparison group. As ALT is the flap of choice for pediatric lower extremity reconstruction, we have a small number of patients, limiting the study's power. Additionally, in this study adult cadavers were used, in which the anatomy might slightly be different than those of pediatric patients. Moreover, the number of cadavers was limited.

## Conclusions

Both the transverse and oblique designs of the DIEP flap are feasible for repairing skin and soft tissue defects of the lower extremities in pediatric patients. Transverse design based on the perforator located below the umbilicus in zone I is recommended for small skin and soft tissue defects. The oblique design of the DIEP flap based on the perforator lateral to the umbilicus in the zone I provides an extended flap for reconstruction of large defects, without insufficient perfusion of the distal part of the flap.

## Data availability statement

The raw data supporting the conclusions of this article will be made available by the authors, without undue reservation.

## Ethics statement

The studies involving human participants were reviewed and approved by Medical Ethics Committee of Xiangya Hospital. Written informed consent to participate in this study was provided by the participants' legal guardian/next of kin. Written informed consent was obtained from a legally authorized representative(s) for anonymized patient information to be published in this article.

## Author contributions

Study conceptualization was performed by JH, MT, and JT. Data collection was performed by JH, HC, GG, PW, FY, and LQ. The first draft was written by JH. Data analysis and review and editing were performed by all the authors.

## Conflict of interest

The authors declare that the research was conducted in the absence of any commercial or financial relationships that could be construed as a potential conflict of interest.

## Publisher's note

All claims expressed in this article are solely those of the authors and do not necessarily represent those of their affiliated organizations, or those of the publisher, the editors and the reviewers. Any product that may be evaluated in this article, or claim that may be made by its manufacturer, is not guaranteed or endorsed by the publisher.
