# Supplementary figures and images for: Strategies for selecting perforator vessels for transverse and oblique DIEP flap in male pediatric patients: Anatomical study and clinical applications
Source: Front Pediatr. 2022 Sep 23;10:978481. doi: 10.3389/fped.2022.978481 (PMC9542642; doi:10.3389/fped.2022.978481)

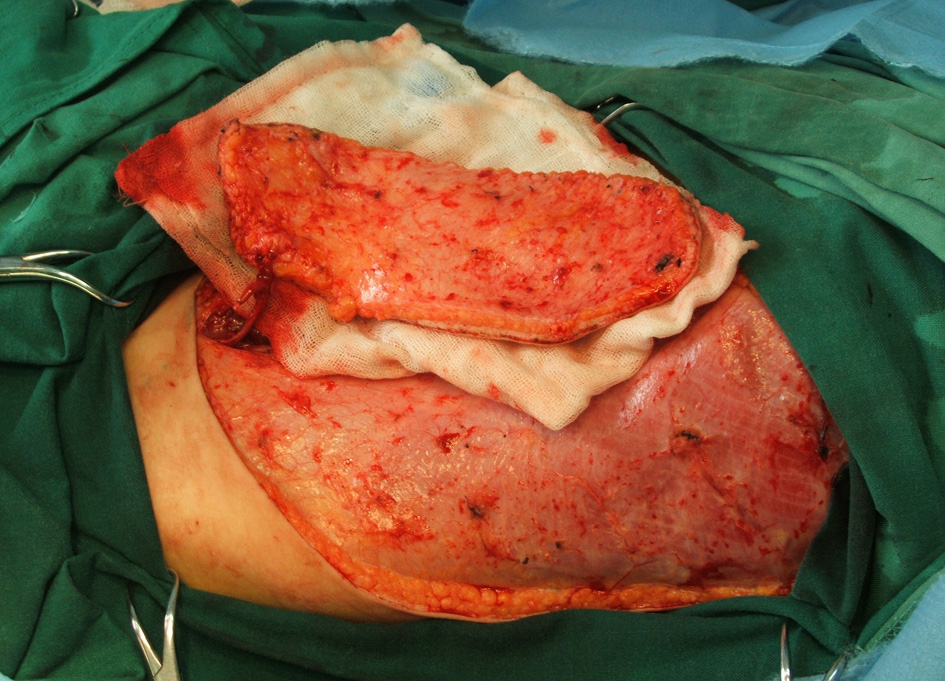

Supplement: Supplementary file 3 [file Image_1.JPEG]

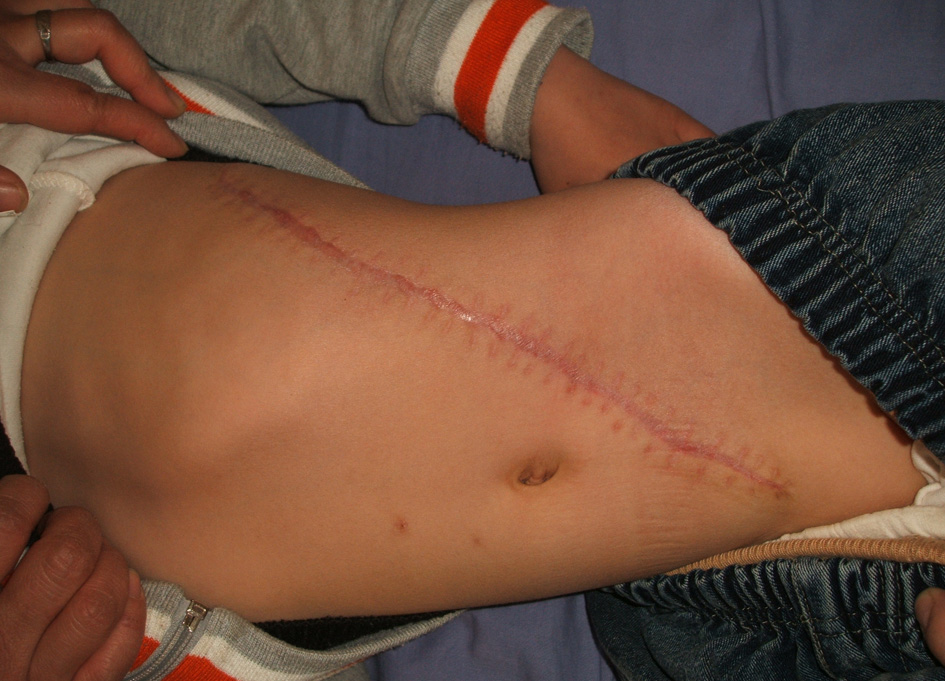

Supplement: Supplementary file 4 [file Image_2.JPEG]
